# Non-pulsatile blood flow is associated with enhanced cerebrovascular carbon dioxide reactivity and an attenuated relationship between cerebral blood flow and regional brain oxygenation

**DOI:** 10.1186/s13054-019-2671-7

**Published:** 2019-12-30

**Authors:** Cecilia Maria Veraar, Harald Rinösl, Karina Kühn, Keso Skhirtladze-Dworschak, Alessia Felli, Mohamed Mouhieddine, Johannes Menger, Ekaterina Pataraia, Hendrik Jan Ankersmit, Martin Dworschak

**Affiliations:** 1 Division of Cardiothoracic and Vascular Anaesthesia and Intensive Care Medicine, Department of Anaesthesia, Intensive Care Medicine, and Pain Medicine, Vienna General Hospital, Medical University of Vienna, Waehringer Guertel 18-20, 1090 Vienna, Austria; 2 Department of Anaesthesia and Intensive Care Medicine, LKH Feldkirch, Feldkirch, Austria; 3 Department of Anaesthesia, Intensive Care Medicine and Pain Medicine, Klinikum Traunstein, Traunstein, Germany; 4 Department of Neurology, Vienna General Hospital, Medical University of Vienna, Vienna, Austria; 5 Division of Thoracic Surgery, Department of Surgery, Vienna General Hospital, Medical University of Vienna, Vienna, Austria

**Keywords:** Cerebrovascular carbon dioxide reactivity, Non-pulsatile blood flow, Regional brain saturation, Cerebral microcirculation, Extracorporeal membrane oxygenation, Non-pulsatile left ventricular assist device, Cerebral blood flow velocity, Extracorporeal cardiopulmonary resuscitation

## Abstract

**Background:**

Systemic blood flow in patients on extracorporeal assist devices is frequently not or only minimally pulsatile. Loss of pulsatile brain perfusion, however, has been implicated in neurological complications. Furthermore, the adverse effects of absent pulsatility on the cerebral microcirculation are modulated similarly as CO_2_ vasoreactivity in resistance vessels. During support with an extracorporeal assist device swings in arterial carbon dioxide partial pressures (PaCO_2_) that determine cerebral oxygen delivery are not uncommon—especially when CO_2_ is eliminated by the respirator as well as via the gas exchanger of an extracorporeal membrane oxygenation machine. We, therefore, investigated whether non-pulsatile flow affects cerebrovascular CO_2_ reactivity (CVR) and regional brain oxygenation (rSO_2_).

**Methods:**

In this prospective, single-centre case-control trial, we studied 32 patients undergoing elective cardiac surgery. Blood flow velocity in the middle cerebral artery (MCAv) as well as rSO_2_ was determined during step changes of PaCO_2_ between 30, 40, and 50 mmHg. Measurements were conducted on cardiopulmonary bypass during non-pulsatile and postoperatively under pulsatile blood flow at comparable test conditions. Corresponding changes of CVR and concomitant rSO_2_ alterations were determined for each flow mode. Each patient served as her own control.

**Results:**

MCAv was generally lower during hypocapnia than during normocapnia and hypercapnia (*p* < 0.0001). However, the MCAv/PaCO_2_ slope during non-pulsatile flow was 14.4 cm/s/mmHg [CI 11.8–16.9] and 10.4 cm/s/mmHg [CI 7.9–13.0] after return of pulsatility (*p* = 0.03). During hypocapnia, non-pulsatile CVR (4.3 ± 1.7%/mmHg) was higher than pulsatile CVR (3.1 ± 1.3%/mmHg, *p* = 0.01). Independent of the flow mode, we observed a decline in rSO2 during hypocapnia and a corresponding rise during hypercapnia (*p* < 0.0001). However, the relationship between ΔrSO_2_ and ΔMCAv was less pronounced during non-pulsatile flow.

**Conclusions:**

Non-pulsatile perfusion is associated with enhanced cerebrovascular CVR resulting in greater relative decreases of cerebral blood flow during hypocapnia. Heterogenic microvascular perfusion may account for the attenuated ΔrSO_2_/ΔMCAv slope. Potential hazards related to this altered regulation of cerebral perfusion still need to be assessed.

**Trial registration:**

The study was retrospectively registered on October 30, 2018, with Clinical Trial.gov (NCT03732651).

## Background

In the field of cardiac surgery and at many ICUs and emergency departments of tertiary care centres, it is daily practice to subject patients to a non-physiological condition where systemic blood flow either completely lacks pulsatility or pulsatility is greatly diminished [1]. Non-pulsatile flow, however, has been linked to increased vascular resistance, raised muscular sympathetic nervous activity, decreased oxygen consumption, endothelial dysfunction with loss of endothelial integrity and deterioration of microvascular perfusion. All of these factors supposedly contribute to increased incidences of gastrointestinal and intracerebral bleeding, strokes, and increased mortality on veno-arterial extracorporeal membrane oxygenation (VA-ECMO) [2–8].

In rats, endothelial-dependent vascular reactivity is dampened in less than 30 min on non-pulsatile cardiopulmonary bypass (CPB) [9]. Furthermore, within such a short period of absent pulsatility, heterogenous flow in human capillaries ensues. It is marked by vessels with absent being closely adjacent to those with very fast perfusion [2]. On the other hand, pulsatile blood flow is associated with improved cerebral microvascular circulation, a more homogenous tissue perfusion, an increased release of nitric oxide (NO) by the vascular endothelium and a higher ATP content in vital organs [4, 10]. Pulsatile CPB flow proved to be advantageous after acute cerebral ischaemia in dogs, in the course of conventional heart surgery as well as in surgery requiring deep hypothermic cardiac arrest [11–13].

Neurological complications such as stroke, bleeding, cognitive dysfunction and delirium still remain a major concern in patients on mechanical circulatory support systems that provide complete non-pulsatile or minimal pulsatile flow (e.g. CPB, VA-ECMO) or continuous flow left ventricular assist devices (CF LVAD) [14–17]. Flow-induced tissue malperfusion, for example, could therefore potentially further augment ischaemia/reperfusion injury in patients in the course of extracorporeal cardiopulmonary resuscitation (ECPR) and loss of pulsatile cerebral perfusion might contribute to worse neurological outcome [4, 7, 18]. In fact, perfused vessel density, an established microcirculatory parameter, was negatively correlated with survival in patients requiring VA-ECMO due to cardiogenic shock [8].

There are a number of factors that determine cerebrovascular myogenic tone and consequently gross cerebral blood flow (CBF) and its local distribution. CBF is regulated by the metabolic demand of the brain, by cerebral perfusion pressure that underlies autoregulation, by the autonomic nervous system, and by hypoxaemia. Arterial partial pressure of carbon dioxide (PaCO_2_) is another pivotal factor. The final effect of each of these CBF modulators results from the intricate interplay with other factors. For a detailed overview on this topic, we would like to refer the reader to recently published in-depth reviews [19, ﻿20﻿]. In brief, changes in PaCO_2_ alter the diameter of cerebral arterioles by ways similar to those operative during changes of pulsatility thereby affecting nutritive blood flow through the capillary bed [10, 21]. Cerebrovascular CO_2_ reactivity (CVR) reflects this ability of the cerebral arterioles to modify CBF by dilating or constricting in response to changes in PaCO_2_ [22].

In instances when there is dual CO_2_ removal (e.g. via the oxygenator on partial cardiopulmonary bypass or ECMO as well as via the respirator), extremes in PaCO_2_ levels are not infrequent. Interestingly in this context, rapid decreases in PaCO_2_ immediately after onset of VA-ECMO treatment were significantly associated with intracerebral bleeds [23]. Although cerebral autoregulation is frequently severely compromised after cardiopulmonary resuscitation (CPR) [24], CVR is still intact and hypocapnia can easily set in especially when shock and therapeutic hypothermia have already diminished CO_2_ production. Hypocapnia post CPR, however, has been associated with poor neurological outcome, at least when it occurred within the first few hours after CPR, which unfortunately is not uncommon [25, 26].

We hypothesised that CO_2_-driven regulation of cerebral oxygenation during non-pulsatile blood flow is altered. Therefore, our objective was to compare the effect of non-physiological non-pulsatile blood flow on CVR and rSO_2_.

## Material and methods

### Study design and population

The present investigation was a prospective case-control study. All procedures were approved by the Ethics Committee of the Medical University of Vienna (No. 117/2008) and were performed with written informed consent from study participants.

Thirty-two adult patients were enrolled in this study. One consenting patient had to be excluded because of an emergency intervention that became necessary prior to commencement of the study. Exclusion criteria were either symptomatic carotid artery disease or luminal narrowing of the carotid artery > 50% as well as the presence of soft plaques without symptoms, neurological pathologies (i.e. history of previous stroke or intracerebral bleeds, obstructive sleep apnea, obesity hypoventilation syndrome, prior meningitis or encephalitis, and multiple sclerosis), COPD with CO_2_ retention or other disturbances of the acid-base balance as well as women of childbearing age. Patients requiring preoperative diagnostic brain imaging studies with symptoms that could be associated with brain pathology were also excluded from the trial.

### Anaesthesia management

All patients received 3.75–7.5 mg midazolam orally 1 h preoperatively. Standard monitoring including a radial artery line was installed in the operating room together with neurological monitoring, i.e. the bispectral index (BIS, Medtronic, Minneapolis, MN, USA) and rSO_2_ determined by near infrared spectroscopy (INVOS 5100C, Covidien, Dublin, Ireland) using two optodes placed over the right and the left forehead, respectively. Monitoring began 10 min before the start of anaesthesia (baseline measurement). Anaesthesia was induced with 2 mg midazolam, 0.2 μg/kg fentanyl and 1–2 mg/kg propofol. Cis-atracurium (0.2 mg/kg) was given for tracheal intubation that was followed by placement of a central venous line. Furthermore, eight patients were additionally monitored with a pulmonary artery catheter.

Subsequently, the middle cerebral artery (MCA) was identified through the transtemporal window by duplex and Colour Doppler. Relative changes in blood flow velocity in this vessel (MCAv) determined by transcranial Doppler (TCD) frequently serve, despite its limitations, as a surrogate of relative changes in CBF [27]. The TCD transducers were screwed tight to a headset to prevent dislocation. Both the left and the right MCA were insonated, and measurements were taken using either both or (if one was distorted) the better signal. CVR [%/mmHg] was calculated for hypo- and hypercapnia as:

$$ \mathrm{CVR}=\left[\left({\mathrm{MCAv}}_{\mathrm{h}}\kern0.5em -{\mathrm{MCAv}}_{\mathrm{l}}\right)/{\mathrm{MCAv}}_{\mathrm{l}}\right]\times 100/\left({\mathrm{PaCO}}_{2\ \mathrm{h}}-{\mathrm{PaCO}}_{2\mathrm{l}}\right) $$

whereby *h* = value determined at the higher PaCO_2_ and *l* = value determined at the lower PaCO_2_ level. The MCAv/PaCO_2_ slope as a measure reflecting the degree of flow velocity changes following corresponding changes in PaCO_2_ was also calculated. In addition, the relationship between relative CBF changes and corresponding proportional shifts in rSO_2_ was determined.

Volume-controlled ventilation was employed before and after CPB. The respective settings were as follows: FiO_2_ 0.3–0.4, tidal volume 7 mL/kg body weight, respiratory rate 9/min, and a PEEP level of 5 mmHg. Respiratory rate and FiO_2_ were adapted to achieve normoventilation and normoxia. Before going off CBP, the trachea was cleared from secretion and at least one vital capacity manoeuvre was performed. Cefazolin (4 g) was administered 30 min before skin incision and 2 g after termination of CPB. Anaesthesia was maintained with 1–2 vol% end-tidal sevoflurane and continuous administration of 200 μg/h fentanyl. BIS values between 40 and 50 were targeted and maintained. An initial dose of heparin (400 IU/kg) was administered before initiation of CPB to achieve an activated coagulation time > 400 s. CPB was primed with 1 L Ringer’s lactate, 500 mL 6% hydroxyethyl starch 130/0.4, 100 mL mannitol 20% and 10.000 IE heparin. On CPB, mean arterial pressure (MAP) and haemoglobin levels were kept constant. At the end of CPB, heparin was reversed by protamine at a 1:1 ratio of the total dose given immediately before CPB.

### Intraoperative measurements (condition 1: non-pulsatile flow)

In order to not delay surgery, we started with the non-pulsatile flow condition. During complete CPB after aortic cross-clamping sweep gas flow at the heart-lung machine was initially set to achieve a PaCO_2_ value of 40 mmHg (the CO_2_ tension can rapidly be determined by in-line measurement at the heart-lung machine after an initial calibration). Subsequently, PaCO_2_ was decreased and increased in a step-wise manner between 30, 40, and 50 mmHg. After a 5-min equilibration period, haemodynamic and metabolic variables were documented for every PaCO_2_ level. At the same time, rSO_2_ was recorded and mean MCAv was measured.

### Postoperative measurements (condition 2: pulsatile blood flow)

Postoperative measurements were performed after ICU admission during stable conditions. Patients received propofol (average dose: 3 mg/kg/h) and remifentanil (0.1 μg/kg/min) to achieve the same BIS values as intraoperatively to ensure a comparable cerebral metabolic rate of oxygen [28]. Volume-controlled ventilation was continued with the abovementioned settings. Minute volume was adapted according to blood gas analysis to reach the desired PaCO_2_ level in the same sequence as on CPB by changing respiratory rate but not tidal volume to avoid concomitant changes in intrathoracic pressure during measurements while we simultaneously tried to maintain sedation, patient positioning, blood pressure, cardiac output and body temperature.

### Statistical analysis

Statistical analysis was performed using Stata/IC 15.1 (StataCorp LLC) and GraphPad Prism 5.04 software (GraphPad Software). Categorical data was reported as count (percentage), normally distributed continuous data as mean (± standard deviation) and non-normally distributed data as median (interquartile range).

The effect of pulsatile and non-pulsatile blood flow on CVR was analysed using multilevel mixed-effects linear regression for repeated measures followed by post hoc Tukey test (xtmixed Stata). A Pearson’s product-moment correlation was utilised to assess the relationship between ΔrSO_2_ and ΔMCAv. A paired *t*-test was performed to analyse differences in MAP, BIS and haemoglobin between both blood flow modalities (non-pulsatile vs. pulsatile). *p* values < 0.05 were considered significant.

## Results

Patient characteristics and type of surgery is depicted in Table 1. For each condition (i.e. pulsatile or non-pulsatile flow), MAP, haemoglobin, BIS, and body temperature did not change over the measurement period. MAP and BIS values, however, were significantly lower on CPB (63 ± 8 vs. 73 ± 10 mmHg and 40 ± 6 vs. 48 ± 7, *p* < 0.0001) while PaO_2_ was significantly higher (175 ± 56 vs. 135 ± 32 mmHg, *p* < 0.05). Isovolaemic haemodilution resulted in a decreased haemoglobin level on CPB (8.4 ± 1.1 g/dL vs. 11.1 ± 1.1 g/dL, *p* < 0.0001). Irrespective of flow mode, we neither observed increases in MAP during hypercapnia (Table 2) nor instances of hypoxaemia.

**Table 1:** Patient characteristics and perioperative data

Gender male/female ratio *n* (%)	23/8 (74/26)
Age (years) mean ± SD	65 ± 8
Arterial hypertension *n* (%)	23 (74)
Chronic obstructive lung disease *n* (%)	4 (12)
Diabetes *n* (%)	5 (16)
Current smoker *n* (%)	10 (32)
Left ventricular ejection fraction *n* (%)	
> 50%	21 (67)
40–50%	4 (12)
< 40%	7 (22)
Height (cm) mean ± SD	172 ± 9
Bodyweight (kg) mean ± SD	82 ± 16
Body mass index (kg/m^2^) mean ± SD	28 ± 5
No antiplatelet therapy *n* (%)	16 (51)
Single antiplatelet therapy *n* (%)	11 (35)
Dual antiplatelet therapy *n* (%)	5 (16)
Surgical procedure *n* (%)	
CABG only	10 (32)
CABG with aortic valve replacement/repair	7 (22)
CABG with mitral valve replacement/repair	7 (22)
Aortic valve replacement	7 (22)
Vasoactive agents	
Dobutamine *n* (%)	11 (35)
Noradrenaline *n* (%)	31 (100)
Levosimendan *n* (%)	5 (16)
Blood and coagulation products	
Packed red blood cells *n* (%)	13 (41)
Fibrinogen *n* (%)	11 (35)
Tranexamic acid *n* (%)	31 (100)
Tranexamic acid (g) mean ± SD	2.5 ± 1.0
Cardiopulmonary bypass time (min) mean ± SD	148 ± 75
Aortic cross-clamping time (min) mean ± SD	93 ± 48
Mild hypothermia *n* (%)	9 (29)
Highest serum lactate level (mg/dL) mean ± SD	2.7 ± 2

*SD* standard deviation, *n* number of patients, *CABG* coronary artery bypass graft

Data are either presented as absolute numbers (percentage) or as means ± SD

**Table 2:** Oxygenation, patient temperature and haemodynamics at the time of measurement

Flow mode PaCO_2_ level	rSO_2_ (%)	SaO_2_ (%)	PaO_2_ (mmHg)	Temp (°C)	MAP (mmHg)	CI (L/min/m^2^)
Non-pulsatile						
30 mmHg	56 [51–60]	99 [98–100]	160 [140–177]	36.3 [35.9–36.7]	63 [57–68]	2.5 [2.4–2.7]
40 mmHg	59 [57–63]	99 [98–100]	170 [154–197]	36.4 [35.8–36.7]	63 [57–69]	2.5 [2.4–2.7]
50 mmHg	62 [57–66]	99 [98–100]	161 [133–190]	36.4 [35.9–36.7]	63 [54–68]	2.5 [2.3–2.7]
Pulsatile						
30 mmHg	58 [56–64]	100* [99–100]	137* [116–161]	36.5 [36.3–36.7]	71* [66–80]	2.7 [2.1–3.3]
40 mmHg	61 [59–67]	100* [100–100]	140* [109–164]	36.4 [36.3–36.7]	72* [65–83]	2.6 [1.9–2.8]
50 mmHg	64 [62–70]	100* [99–100]	120* [101–146]	36.5 [36.3–36.7]	68* [65–75]	2.6 [2.0–3.3]

Values are medians [25th–75th percentile], *rSO*_*2*_ Regional brain saturation, *SaO*_*2*_ Arterial haemoglobin oxygen saturation, *PaO*_*2*_ Arterial partial pressure of oxygen, *Temp* patient temperature *MAP* mean arterial blood pressure, *CI* cardiac index (*n* = 8), * = *p* < 0.05 vs. corresponding value during non-pulsatile flow (ANOVA with post hoc Tukey test)

**Fig. 1:**
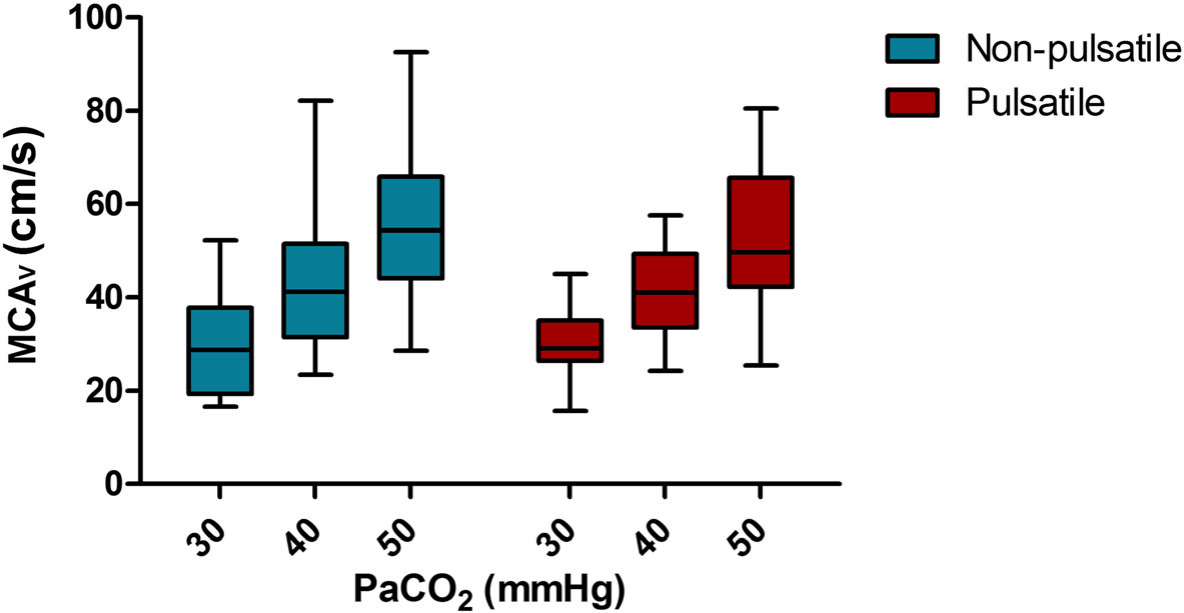
Absolute MCAv measures determined during non-pulsatile and pulsatile flow at hypo-, normo-, and hypercapnia *MCAv* blood flow velocity in the middle cerebral artery

**Fig. 2:**
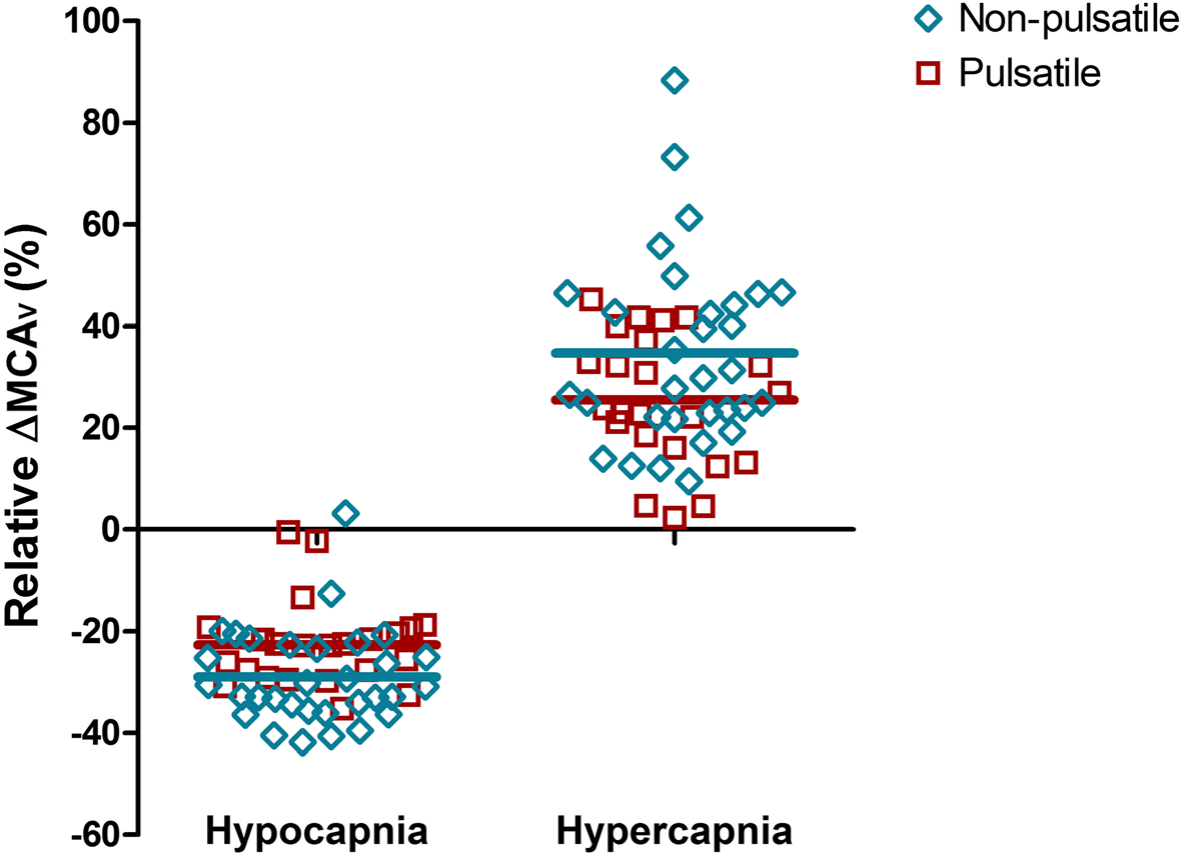
Relative MCAv changes of single patients after induction of hypo- and hypercapnia *ΔMCAv* relative change in blood flow velocity in the middle cerebral artery

Absolute values of rSO_2_, arterial haemoglobin oxygen saturation, PaO_2_, patient temperature, MAP, and, where available, intra- and postoperatively determined cardiac indeces after CO_2_ step changes during non-pulsatile and pulsatile flow are listed in Table 2. The course of absolute MCAv during alterations of PaCO_2_ within each flow mode is depicted in Fig. 1. MCAv was significantly lower during hypocapnia compared to normocapnia (*p* < 0.0001), while MCAv was significantly higher during hypercapnia compared to normocapnia (*p* < 0.0001). There was also a statistically significant difference between flow modes (*p* < 0.033). Relative MCAv changes of individual patients clearly show that greater shifts from normocapnia occurred when flow was non-pulsatile (Fig. 2, *p* < 0.05). The MCAv/PaCO_2_ slope was 14.4 cm/s/mmHg [CI 11.8–16.9], during non-pulsatile and 10.4 cm/s/mmHg [CI 7.9–13.0] during pulsatile blood flow (*p* = 0.032).

**Fig. 3:**
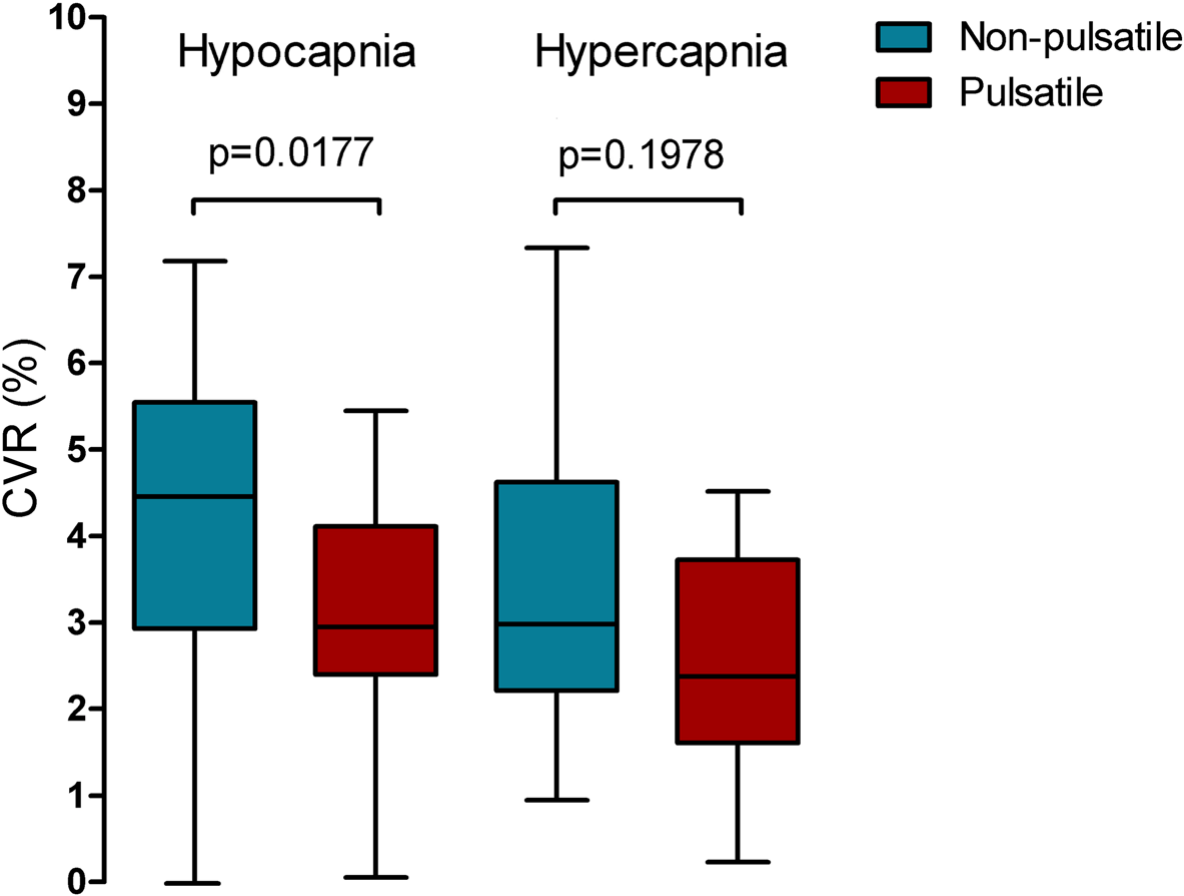
Cerebrovascular CO_2_ reactivity during hypo- and hypercapnia and pulsatile as well as non-pulsatile blood flow *CVR* cerebrovascular CO_2_ reactivity

Under hypocapnia and non-pulsatile flow, CVR was significantly increased (4.3 ± 1.7%/mmHg) compared to pulsatile flow (3.1 ± 1.3%/mmHg, *p* = 0.017). During hypercapnia, however, non-pulsatile CVR was only nominally but not statistically greater in relation to pulsatile CVR (3.5 ± 1.8%/mmHg vs. 2.6 ± 1.2%/mmHg, *p* = 0.197, Fig. 3).

**Fig. 4:**
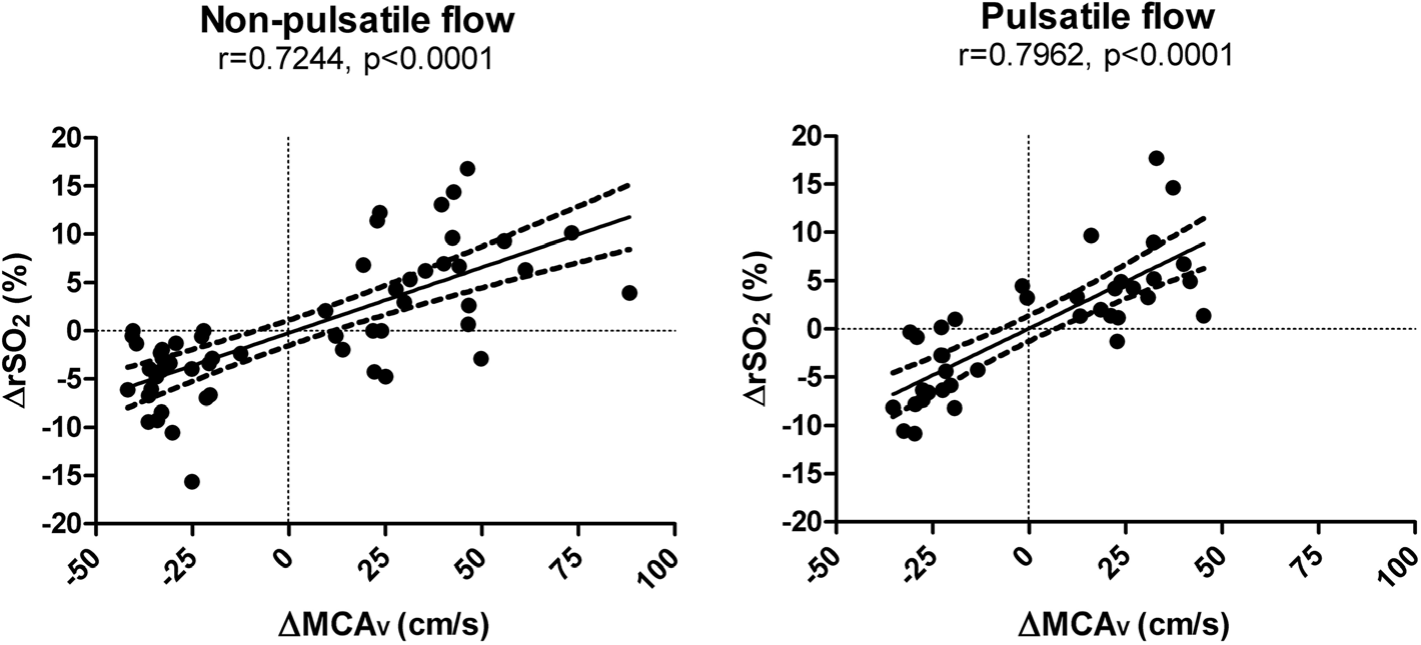
Correlation of ΔMCAv and ΔrSO_2._ *r* correlation coefficient, *ΔMCAv* relative change in blood flow velocity in the middle cerebral artery, *ΔrSO*_*2*_ relative change in regional cerebral oxygen saturation

Independent of the flow mode, we saw a decline in rSO_2_ during hypocapnia and a corresponding rise during hypercapnia (*p* < 0.0001). However, despite more pronounced CO_2_-driven flow velocity changes during non-pulsatile flow, there was no equivalent difference in ΔrSO_2_ between corresponding PaCO2 levels when compared to pulsatile flow. The rSO_2_/PaCO_2_ slope was 2.8%/mmHg [CI 1.5–4.0] on CPB and 3.1%/mmHg [CI 1.9–4.3], postoperatively (*p* = 0.72). As shown in Fig. 4, ΔMCAv and ΔrSO_2_ changes were closely related to each other during non-pulsatile (*r* = 0.724, *p* < 0.001) and pulsatile blood flow (*r* = 0.796, *p* < 0.001). However, during non-pulsatile flow, the slope of the regression line was less steep and cerebral CVR more accentuated as compared to pulsatile perfusion.

## Discussion

In the present study, short-term non-pulsatile perfusion was associated with significantly enhanced cerebrovascular CVR compared to pulsatile blood flow resulting in exaggerated responses in CBF when non-physiologically low PaCO_2_ values were reached. These changes in CBF, however, were not accompanied by equivalent changes in rSO_2_.

Instances of hypocapnia are not infrequent in low cardiac output and INTERMACS 1 or 2 patients on extracorporeal assist devices with or without dual CO_2_ removal (e.g. VA-ECMO, extracorporeal cardiopulmonary resuscitation (ECPR) or CF LVAD) as well as after conventional CPR [26]. They have mostly been associated with poor neurological outcome. Prolonged intraoperative hypocapnia by itself has recently also been associated with postoperative delirium in non-cardiac surgery [29]. Our observations indicate that the effect of hypocapnia-induced cerebral vasoconstriction may even be more extensive in the presence of non-pulsatile cerebral blood flow. In contrast, the impact of hypercapnia on vasodilation was not significantly different from that seen under pulsatile flow conditions in the current study.

To guarantee propagation of pulsatility into the cerebral circulation, we compared CVR during non-pulsatile blood flow on complete CPB with the same patient’s CVR postoperatively on the ICU during pulsatile blood flow generated by the beating heart itself. In previous studies investigating induced pulsatile flow on CPB, it has never been ruled out that it was simply blunted pulsatility (i.e. with reduced systolic and pulse pressures) that was created, which lacks excess energy [30]. Moreover, even in the paediatric population, not all available pulsatile pumps generate this higher haemodynamic energy when compared with non-pulsatile pumps [31]. Mitigated pulsatility, however, which is frequently observed in ECMO patients early after implant, appears to be similar to non-pulsatile flow as it also increases muscular sympathetic nerve activity [32]. Furthermore, long duration of diastole and low levels of diastolic pressure elicited sympathetic bursts in CF LVAD as well as in VA-ECMO patients with minimal pulsatility [32]. Interestingly in this context, 2 h after exposure to VA-ECMO with a high flow rate (120–150 mL/kg/min), decreased NO production was noted in cerebral arteries of lambs indicating impaired cerebral vascular endothelial function [33]. This phenomenon likely contributes to capillary collapse and shunting when a critical closing pressure cannot be reached whereby the driving pressure is usually created by the excess energy generated through the ejection of blood from the left ventricle into the aorta [30]. O’Neil and co-workers studied sublingual microcirculation on CPB, yet without specifically looking into PaCO_2_-generated vasoreactivity and found that the proportion of normally perfused microvessels decreases immediately with onset of non-pulsatile flow [2]. In a similar study in 20 patients undergoing cardiac surgery, the same researchers reported an increase in vessels with hyperdynamic flow when blood flow was non-pulsatile, which may represent capillary shunting [5]. These results could account for our observation that despite greater ΔMCAv during non-pulsatile flow, ΔrSO_2_ did not change proportionally although a close relationship still persisted. Conversely, this also implies that potential benefits of pulsatile flow related to improved microcirculatory perfusion may not be traceable by higher rSO_2_ values [34]. The dependence of brain tissue saturation on PCO_2_ has previously been confirmed in healthy individuals and patients undergoing cardiac surgery [35]. In addition, during carotid endarterectomy, rSO_2_ closely correlated with MCAv during cross clamping of the ipsilateral common carotid artery [36]. These findings are in line with our results as rSO_2_ and PaCO_2_ changed in the same direction. rSO_2_ further changed in parallel to MCAv. However, there appears to be some uncoupling during non-pulsatile flow with enhanced reductions in cerebral blood flow during hypocapnia.

Findings from previous studies in this area can barely be compared with our results since investigations were then usually conducted with only few patients assigned to either of the two flow modes, during moderate hypothermic CPB, with different anaesthetics (e.g. N_2_O), and partly at very low cerebral perfusion pressures [37]. In our trial, the 31 patients served as their own controls and only few patients were operated under mild hypothermia. In addition, several studies have shown that CVR is maintained with both anaesthetics we employed when given within normal clinical ranges [38] as was the case here, despite the fact that anaesthesia likely compromises basal vascular tone [39]. In this trial, end-tidal sevoflurane concentrations ranging between 0.3 and 1.5 MAC (mean dose 0.8 MAC) was administered intra- and low-dose propofol (median 2.5 [range 1.8–3.9] mg/kg/h) postoperatively.

Although changes in PaCO_2_ alter the diameter of cerebral arterioles and thus determine nutritive microcirculatory blood flow through the capillary bed, the large cerebral arteries are frequently considered to be noncompliant serving as a conduit to distribute and store blood volume [27]. In young, healthy, and spontaneously breathing volunteers, however, luminal changes of up to 10% have been calculated during hypo- and hypercapnia when ΔPetCO_2_ exceeded 10 mmHg [40]. It is unclear if similar changes also occur in sick, anaesthetised and ventilated patients undergoing cardiac surgery. As we did not measure the diameter of the interrogated vessel, we cannot rule out that variations in the vessel calibre impacted on the measured MCAv whereby particularly MCAv values during hypercapnia would be affected. The modest hyperoxia during non-pulsatile flow on CPB should rather decrease the slope of the curve describing the relationship between PaCO_2_ and MCAv, which would be just the opposite we found [41]. As mentioned above, sevoflurane anaesthesia that we used on CPB has been demonstrated to preserve CVR (4.1 ± 0.7%/mmHg PaCO_2_) as well as MCAv in healthy patients undergoing orthopaedic surgery when MAP was kept at 80 mmHg with phenylephrine [42]. MAP in our patients was generally < 80 mmHg and showed minor though statistically significant differences between pulsatile and non-pulsatile flow. In this regard, it should, however, be kept in mind that current research confirmed interactions between CVR and cerebrovascular autoregulation, primarily affecting the upper threshold [43, 44]. Nevertheless, MAPs we recorded were all within the critical PaCO_2_-dependent autoregulatory thresholds that have been reported during anaesthesia [37, 45]. In our trial, cerebral perfusion pressures thus should not have had an effect on the measured MCAv. Neither during non-pulsatile nor during pulsatile flow did we observe rises in MAP accompanying periods of hypercapnia. A potential explanation may be the suppression of sympathetic activity during anaesthesia. Interestingly in this context, as CVR is also mitigated during sympathetic blockade [46, 47], increased sympathetic activity in turn, which has been confirmed during non-pulsatile blood flow [48], might augment CVR, which is exactly what we have seen. It is, therefore, probably not pulsation per se that accounts for the observed changes in the PaCO_2_-dependent regulation of cerebral artery myogenic tone [44] but secondary effects induced by pulsatile blood flow like for example eNOs activity and coupling [49, 50] or modulations of the autonomic nervous system output [51]. Furthermore, a reduced myogenic tone has been observed in isolated murine cerebral arteries when subjected to static pressure, which may also account for the accentuated CVR we determined [50].

## Limitations

This trial still does have some limitations primarily related to the chosen protocol. Use of CPB always causes some degree of isovolaemic haemodilution that may impact blood viscosity and eventually microcirculatory perfusion. Haemodilution might thereby affect the magnitude of CVR. Data from the literature, however, is inconclusive. At haematocrits < 28%, both attenuated and enhanced CVR has been reported in patients undergoing heart surgery during hypocapnia, while no alteration was seen during hypercapnia [52, 53]. However, isovolaemic haemodilution by low-molecular-weight dextran infusion, for example, did not increase but instead decreased acetazolamide-induced cerebral vasoreactivity [54]. In addition, CBF is augmented when arterial oxygen content drops, which, however, should only have a marginal effect considering the actual haemoglobin levels in our patients during and after surgery [55]. Nevertheless, the enhanced CVR we observed under non-pulsatile flow may be related to some extent to the lower haematocrits on CPB.

Another issue that needs to be addressed is the different types of anaesthetics that were employed here as their impact on the BIS level, CBF, and CMRO_2_ may not be the same. Sevoflurane was administered intraoperatively due to its ascribed cardioprotective properties but could not be continued into the postoperative period as we lack the technology that allows its administration in the ICU. Data from eight young and healthy volunteers indicate that 1.5 vol% sevoflurane reduces regional CBF less than propofol (propofol plasma concentration ranged between 2.6 and 4.6 μg/mL) whereby CMRO_2_ was equally decreased and MAP and BIS depressed to a similar degree [56]. In relation, the propofol dose our patients received was much less due to the concomitant administration of remifentanil, which may explain the greater neuronal activity in the frontal cortex evidenced by higher BIS levels postoperatively. Therefore, one should not expect to find a significant propofol-induced depression of global CBF although we cannot comment on the eventual regional distribution of CBF. In this context, alterations in the cortical CBF/CMRO_2_ ratio have only been observed at higher propofol concentrations (i.e. 7.8 ± 2.1 mg/kg/h) that were also accompanied by a BIS level as low as 20, which makes it unlikely that propofol in this study affected postoperative rSO_2_ [57]. As a matter of fact, mean MCAv under 6 mg/kg/h propofol anaesthesia (a higher dose than we employed) observed in ASA 1 patients was by trend somewhat higher but comparable with MCAv of our patients in the ICU (41–49–58 cm/s for hypo-, normo-, and hypercapnia). In addition, relative CVR was preserved in this investigation [58]. In comparison, equivalent MCAv measured again in young and healthy ASA 1 patients, however, under 1.2 and 1.5 MAC sevoflurane anaesthesia were 25/33–47/55–76/78 cm/s [59, 60], which are strikingly similar to those obtained in a spontaneously breathing non-anaesthetized young volunteer (30–50–70 cm/s) [41]. Obviously, mean absolute MCAv can vary to some extent. Hence, relative CVR seems to be a more appropriate indicator of the effect of PaCO_2_ alterations on CBF as absolute measures.

Despite the mentioned drawbacks of our protocol, it does have advantages, too. Because we did not compare different groups of patients subjected to different flow modes, we could reduce differences in ΔMCAv resulting from other potential confounders (e.g. diabetes mellitus, arterial hypertension, age, atherosclerosis of the cerebral vessels, previously unrecognised anatomical abnormalities). Furthermore, it seems improbable that CVR was affected by mild hypothermia with α-stat pH management that was instituted in few patients on CPB [52]. Moreover, none of the patients had developed a fever when the second measurement was carried out in the ICU. Additionally, catecholamines as well as levosimendan were administered at low dosages that should not affect CVR under anaesthesia to a meaningful extent [61, 62]. They were frequently already started intraoperatively and continued after surgery.

## Conclusion

In conclusion, in older anaesthetized cardiac surgery patients, non-pulsatile blood flow caused enhanced CVR. If, however, unphysiological PaCO_2_ values in conjunction with this flow mode actually increase the odds of undesired cerebral complications, as it was the case after traumatic brain injury, requires further investigations [63]. Especially with a leftward-shifted oxygen-haemoglobin dissociation curve and a heterogenous microvascular perfusion, rSO_2_ recordings may additionally overestimate true brain tissue oxygenation during hypocapnia when CBF becomes non-pulsatile. This would further diminish the prognostic value of rSO_2_ post ECPR regarding neurological outcome [64].

As even short-term non-pulsatile flow conditions rapidly affect the cerebral circulation, these findings could be especially relevant in patients requiring extracorporeal mechanical assist devices that do generate either no or merely blunted pulsatile brain perfusion. Patients with low left ventricular ejection fraction receiving emergent VA-ECMO and depicting low-pulsatile systemic perfusion could potentially be particularly vulnerable regarding cerebral insults during hypothermic and hypocapnic states [3, 65, 66]. The currently poor neurological outcome after cardiac arrest with or without ECPR is often clinically initiated by convulsions and myoclonus. In this context, hypocapnia-induced vasoconstriction leading to tissue alkalosis and hypoxaemia might also play a certain role in the elicitation of seizures in susceptible patients immediately after global brain ischaemia [67–69].

## Data Availability

The datasets used and/or analysed during the current study are available from the corresponding author on reasonable request.

## Abbreviations

BIS: Bispectral index
CBF: Cerebral blood flow
CF LVAD: Continuous flow left ventricular assist device
COPD: Chronic obstructive pulmonary disease
CVR: Carbon dioxide reactivity
CPB: Cardiopulmonary bypass
CPR: Cardiopulmonary resuscitation
ECMO: Extracorporeal membrane oxygenation
ECPR: Extracorporeal cardiopulmonary resuscitation
ICU: Intensive care unit
MAP: Mean arterial pressure
MCA: Middle cerebral artery
PaCO_2_: Arterial partial pressure of oxygen
PEEP: Positive end-expiratory pressure
rSO_2_: Regional brain tissue saturation
TCD: Transcranial Doppler
VA-ECMO: Veno-arterial extracorporeal membrane oxygenation
MCAv: Blood flow velocity determined in the middle cerebral artery
